# Development of an artificial foot enabling the simulation of the natural behaviour of the human unroll of the foot during walking and running

**DOI:** 10.1186/1757-1146-5-S1-O3

**Published:** 2012-04-10

**Authors:** Helga Vertommen, Eveline De Raeve, Wim Dewindt, Carel Van den Bosch, Fred Holtkamp, Louis Peeraer

**Affiliations:** 1 MOBILAB, University College Kempen, Geel, Belgium; 2 Greentech Engineering, Eindhoven, The Netherlands; 3 Faculty of Kinesiology and Rehabilitation Sciences, KULeuven, Leuven, Belgium; 4 Fontys University of Applied Sciences, Eindhoven, The Netherlands

## Background

The percentage of sports and leisure shoes sold worldwide is gradually increasing. However, consumers have little or no objective information on the mechanical properties of the shoes. A justified selection protocol of sports and leisure shoes based on static and dynamic shoe properties considering the intended use is essential. Today, commonly accepted dynamic test protocols for (sports) shoes do not exist.

The development of an artificial parametric foot as part of an innovative robot gait simulator is a tool to objectify shoe properties independently from possible compensations encountered during assessment of test persons. This contribution discusses the development of an artificial foot enabling objective testing of the mechanical and functional properties of sports and leisure shoes.

## Materials and methods

**Figure 1:**
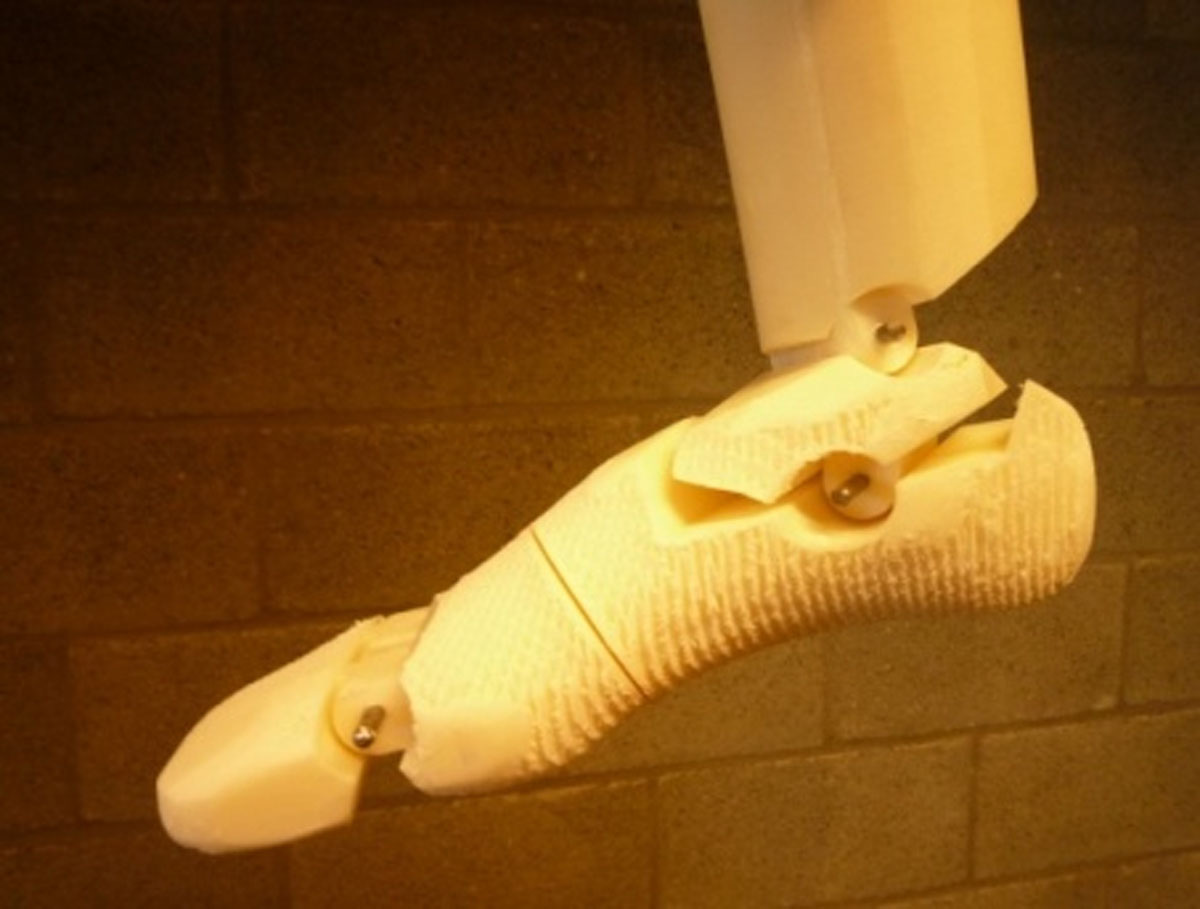
Initial design of the artificial foot

An artificial foot consisting of the shank, the rear foot, the mid foot and the toes was designed based on a measurement protocol for barefoot running using 12 active markers sampled at 400 Hz [1]. The foot was manufactured using additive layered fabrication (Figure 1).

## Results

**Figure 2:**
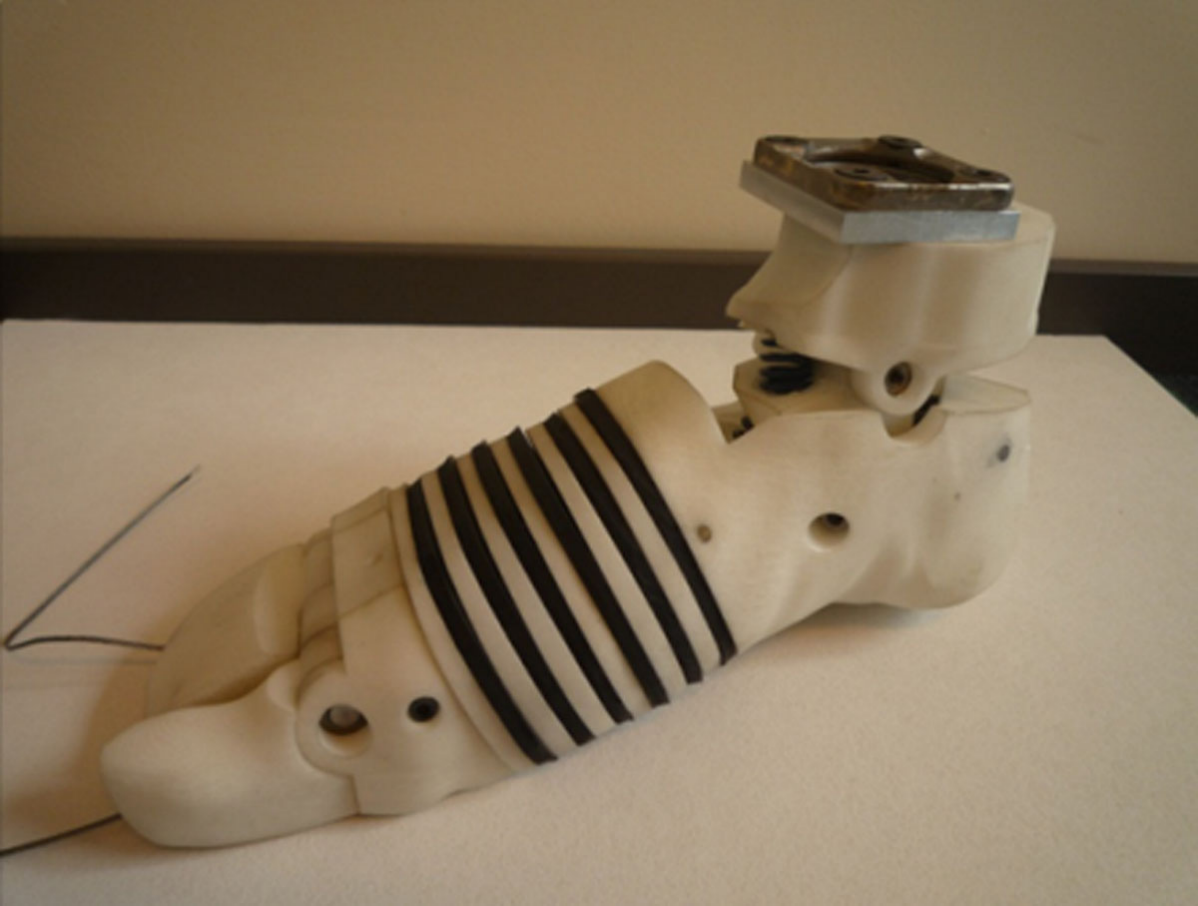
Second design of artificial foot

Based on measurements the design of this initial foot was optimized with respect to functionality and appropriate shoe fit. The multi segment design of this foot is shown in figure 2. Optimisation was realized by changing 3 different regions: (a) orientation of the subtalar joint axis, (b) segmentation of the mid foot into 6 different slices matching realistic functional behaviour during roll off (c) segmentation of the toe section into two parts (big toe and lesser toes) to simulate a better forefoot roll off. Foot segments are connected with springs and dampers, each of them designed on literature findings and model simulations.

## Conclusions

An improved artificial foot design with built-in springs and dampers for objective testing of shoe properties is realized. The design needs further elaboration with respect to geometry, foot axes and functional behaviour. This mechanical foot is a promising tool to help us understand mechanical properties of shoes using strictly standardised testing protocols using mechanical gait simulators.
